# MicroRNAs in Insulin Resistance and Obesity

**DOI:** 10.1155/2012/484696

**Published:** 2012-07-18

**Authors:** Michael D. Williams, Geraldine M. Mitchell

**Affiliations:** ^1^O'Brien Institute, Melbourne, VIC 3065, Australia; ^2^Department of Surgery, University of Melbourne, St Vincent's Hospital, Melbourne, VIC 3065, Australia; ^3^Faculty of Health Sciences, Australian Catholic University, Melbourne, VIC 3065, Australia

## Abstract

MicroRNAs (miRNAs) are a class of short, single-stranded non-protein coding gene products which can regulate the gene expression through post-transcriptional inhibition of messenger RNA (mRNA) translation. They are known to be involved in many essential biological processes including development, insulin secretion, and adipocyte differentiation. miRNAs are involved in complex metabolic processes, such as energy and lipid metabolism, which have been studied in the context of diabetes and obesity. Obesity, hyperlipidemia (elevated levels of blood lipids), and insulin resistance are strongly associated with the onset of type 2 diabetes. These conditions are also associated with aberrant expression of multiple essential miRNAs in pancreatic islets of Langerhans and peripheral tissues, including adipose tissue. A thorough understanding of the physiological role these miRNAs play in these tissues, and changes to their expression under pathological conditions, will allow researchers to develop new therapeutics with the potential to correct the aberrant expression of miRNAs in type 2 diabetes and obesity.

## 1. Introduction

Type 1 diabetes is characterized by the selective autoimmune-mediated destruction of the *β* cells in the islets of Langerhans in endocrine pancreas. This pathological loss of *β*-cell mass in the pancreas results in a failure to produce insulin in response to changes in blood glucose levels. Sufferers of type 1 diabetes require stringent monitoring of blood glucose levels and treatment with exogenous insulin administered through regular injections or through continuous monitoring insulin pumps, to replace the absent insulin. Type 2 diabetes is a less defined condition, culminating in dysregulation of blood glucose levels but caused by the development of insulin resistance and/or relative insulin deficiency. In this context, the body may maintain its ability to secrete insulin; however, the biochemical signalling required for the secretion of insulin or the sensitivity of cells to the released insulin is deficient, resulting in a blunted response and aberrant blood glucose levels. Early detection provides the opportunity for the disease to be managed using life-style changes; however, disease progression and eventual reduction in *β*-cell mass require insulin supplementation, similar to the pathology of type 1 diabetes. Obesity, hyperlipidemia (elevated levels of blood lipids) and insulin resistance are strongly associated with the onset of type 2 diabetes. Excessive weight strongly correlates with disease progression and increased risk of detrimental complications. Type 2 diabetes and obesity are considered to be so closely related that they are sometimes referred to collectively as “diabesity” [1].

MicroRNAs (miRNAs) are a class of short, single-stranded non-protein coding gene products typically 20–22 nucleotides in length. They are present in the genomes of eukaryotic organisms and known to post-transcriptionally regulate the expression of target genes through interactions with specific mRNAs [2, 3]. miRNAs are known to negatively regulate gene expression at the post-transcriptional level, either by inhibiting translation or by degrading the target mRNA [4, 5]. This is achieved through interactions between the miRNA seed sequence (first 2 to 8 bp of miRNA) and the 3′-untranslated regions (3′-UTRs) of specific mRNAs. The nature of these interactions affords an individual miRNA with the potential to regulate multiple genes across the diverse eukaryote genome. Such interactions have been shown to regulate multiple diverse biological processes including development [6, 7], insulin secretion [8], neurodevelopment [9] and cell differentiation [10].

Following the discovery that miRNAs were involved in many relevant biological processes, including energy and fat metabolism, these molecules have received growing attention in the fields of diabetes and obesity research. Through the understanding of the role miRNAs play in normal physiology and pathological conditions, researchers will be able to unlock a new class of therapeutic targets with the potential to significantly alter multiple aspects of their respective conditions.

## 2. miRNAs in Pancreatic **β** Cells

Islets of Langerhans are highly-vascularized microorgans, located within the pancreas, primarily responsible for the maintenance of normal blood glucose concentration [11]. Blood glucose levels are maintained within a narrow reference ranged between 3.6 mM and 5.7 mM (mmol·L^−1^) in humans as part of metabolic homeostasis. Glucose homeostasis is primarily achieved through the antagonistic actions of the islet hormones insulin and glucagon, released from islets. Each islet is composed of multiple cell types characterized by the endocrine hormones they produce. These include insulin-producing *β* cells, glucagon-producing *α* cells, somatostatin-producing *δ* cells, ghrelin-producing *ε* cells, and pancreatic polypeptide-producing (PP) cells [12, 13]. During embryonic development, the pancreatic primordial bud is derived from the posterior foregut endoderm. Endocrine, exocrine, and ductal structures subsequently differentiate through a complex branching morphogenic process [14].

miRNAs have been found to be important for the proper development of the pancreas, in particular the insulin-producing *β*-cell population. A conditional deletion of Dicer [15], a cleavage protein essential in miRNA biogenesis resulting in total ablation of all miRNA transcripts in pancreatic progenitor cells in a mouse model produced significant defects in all resulting pancreatic cell lineages. Endocrine cells, in particular the *β*-cell population, were the most dramatically reduced. This effect on endocrine cells was attributed to increased Hes1 expression and consequent reduction in the key pancreatic transcription factor ngn3 (neurogenin-3) during development. The effects of miRNA-mediated regulation of ngn3 activity have been further studied by Joglekar et al. [10], who demonstrated that following 70 percent pancreatectomy, the expression pattern of pancreatic transcription factors upstream of ngn3 was similar to that observed during developing and regenerating pancreas. However, ngn3 (and downstream transcription factors neuroD and nkx2.2) was not detected during regeneration and determined to be post-transcriptionally silenced by miRNAs, including miR-15a, miR-15b, miR-16, and miR-195. The elevated expression of these miRNAs in regenerating pancreas was theorised to facilitate ngn3 independent endocrine regeneration. These studies demonstrate the capacity for miRNAs to significantly alter the behaviour of endocrine cell populations through regulation of key endocrine pancreatic transcription factors under different conditions.

Many genes expressed in pancreatic *β* cells are differentially regulated in real time with respect to changes in blood glucose levels to optimise the production and secretion of insulin [16]. The functional release of insulin, the transcription and stability of insulin mRNA, insulin translation and insulin processing are all regulated by glucose concentrations in *β* cells. The abundance of several miRNA transcripts is also known to change in response to altered glucose concentrations. Tang et al. [17] demonstrated that exposing cultured pancreatic *β*-cell line (MIN6) to simulated prolonged high-glucose conditions could significantly alter the expression of a large number of miRNAs. miR-124a, miR-107 and miR-30d were upregulated and miR-296, miR-484 and miR-690 were downregulated in persistent high glucose conditions. Increased expression of miR-30d in high glucose conditions correlated with increased insulin gene expression but no associated increase in insulin secretion, suggesting that targets of miR-30d are negative regulators of insulin gene expression, independent of insulin secretion. However, a number of other miRNAs have been identified as having direct effects on the various subcellular events involved in glucose-stimulated insulin secretion (GSIS). Hennessy et al. [18] characterized the loss of GSIS in glucose nonresponsive MIN6 cells and discovered that a panel of ten miRNAs (miR-369-5p, miR-130a, miR-27a, miR-410, miR-200a, miR-337, miR-532, miR-320, miR-192 and miR-379) was differentially expressed in non-responsive cultures, implicating these miRNAs in GSIS. 

In 2004, Poy et al. [8] cloned 11 novel miRNAs from pancreatic endocrine cell lines. This study revealed that miRNAs were not only involved in regulating pancreas development but were also involved in other vital biological processes including insulin secretion. This study also identified miR-375, an evolutionarily-conserved islet-specific miRNA, which has since become one of the best characterized islet-specific miRNAs implicated in both insulin secretion [8] and glucose homeostasis [19]. miR-375 was initially implicated in insulin release and was able to negatively regulate GSIS with no effect on either ATP production or intracellular calcium levels. miR-375 was experimentally shown to act at a late step of insulin exocytosis to suppress insulin release. Mtpn (myotrophin), a protein implicated in actin depolymerization and vesicular fusion, was predicted to be a target for miR-375. This was experimentally validated, with inhibition of mtpn production recapitulating the effects of miR-375 on insulin secretion. Mtpn also acts in the nucleus as a transcription factor to activate nuclear factor *κ*B (NF-*κ*B), a critical component in maintaining GSIS in *β* cells [20, 21].

Isolated islets and cell lines exposed to high glucose demonstrate decreased expression of miR-375 which correlated with increases in pdk1 (3′-phosphoinositide-dependent protein kinase 1) and insulin gene expression [22]. Pdk1 is an important component of the pi3k/protein kinase B signal cascade responsible for the growth and developmental effects of insulin [23]. Loss of pdk1 in *β* cells results in progressive hyperglycemia due to reductions in islet density as well as in the number and size of endocrine cells [24]. Pdk1 was later identified as a direct target of miR-375. Overexpression of miR-375 resulted in reduced *β*-cell number, viability and reduced sensitivity to glucose-stimulated insulin transcription, all of which are reversed with inhibition of miR-375. Hyperglycemia is similarly observed in miR-375 knock-out animals, which is also attributable to reduced *β*-cell mass [19]. In these knock-out animals, the important negative growth regulators modulated by miR-375 were found to be upregulated. The condition was more pronounced in animals subjected to metabolic stress, indicating miR-375 plays a crucial role in *β*-cell compensation when metabolic demand is increased. 

Plaisance et al. [25] demonstrated miR-9, a miRNA expressed in pancreatic *β* cells in rat and mouse models, was able to regulate insulin release in *β* cells through interactions with the transcription factor oc2 (onecut-2). Oc2 negatively regulates granuphilin (also known as SLP4/SYTL4), a Rab GTPase effector associated with *β* cell secretory granules. Basal expression of miR-9 is essential for maintaining appropriate granuphilin levels and optimal insulin secretory capacity in *β* cells. However, elevated levels of miR-9 results in reduced GSIS in affected cells. Granuphilin-null mice also show impaired GSIS, with *β*-cells containing fewer insulin granules at the membrane and increased insulin release in response to glucose stimulation [26].

miRNAs have the potential to affect insulin release through other mechanisms, including the expression of the insulin gene. miR-124a exists in three isoforms (miR-124a1, miR-124a2, and miR-124a3). miR-124a2 is significantly upregulated in late pancreas development (e18.5), coinciding with a critical stage in *β*-cell differentiation. This isoform was found to target foxA2 (forkhead/winged helix transcription factor boxa2) and creb-1 (cAMP-response-element binding protein) mRNA. FoxA2 is an upstream regulator of pdx1 (pancreatic duodenal homeobox 1) [27]. Pdx1 is essential for glucose homeostasis and pancreas development and together with ngn3 and mafA represent the key transcription factors essential for *β*-cell differentiation. When pdx1, ngn3, and mafA are re-expressed in an experimental context, these three primary factors are capable of inducing differentiation of unrelated cell types into functional insulin-producing, islet-like structures, indistinguishable from endogenous islet *β* cells capable of ameliorating hyperglycaemia [28, 29]. Foxa2 is an essential activator of genes that function in multiple pathways responsible for insulin secretion. Through the regulation of foxA2, miR-124a can modulate insulin expression indirectly through pdx1 [30]. It can also regulate K_ATP_ channel subunits sur1 (sulphonylurea receptor 1) and kir6.2 (inward rectifier K+ channel member 6.2) [31], both of which are essential for regulated insulin release but have little functional effect on GSIS [30]. However, miR-124a has been shown to target other aspects of the exocytotic machinery responsible for insulin release, including rab27A, snap25, rab3A, syn1 (synapsin 1A) and noc2 (nucleolar complex associated 2) which are involved in GSIS [32].

## 3. miRNAs in Energy Metabolism

In 2005, Mersey et al. [33] provided the first evidence of the use of a miRNA to exert control on a metabolic pathway in mammals. This discovery provided evidence that miRNAs were involved in complex metabolic processes, such as energy metabolism, which has since been studied in the context of diabetes and adipose tissue biology. Glucose homeostasis requires both the presence of insulin and sensitivity of the target tissues to elicit the necessary response. The aetiology of type 2 diabetes is not fully understood; however, insulin resistance is a persistent fundamental finding in patients, and this usually manifests years before the disease is diagnosed clinically. Insulin resistance is considered the best predictor of type 2 diabetes [34]. Insulin resistance is defined as an inability to properly respond to either endogenously produced or exogenously administered insulin in peripheral tissues, such as adipose, liver and skeletal muscle tissues. This results in impaired insulin-mediated glucose uptake in the skeletal muscle and adipose tissues, and insulin-mediated suppression of hepatic glucose output. The resulting insulin insufficiency and chronic hyperglycemia progress to type 2 diabetes. The role of miRNAs in the physiological and pathological function of adipose tissue has received increased attention, particularly given their roles in glucose and lipid metabolism. 

The mechanism responsible for insulin resistance is not fully understood but links between insulin resistance, dyslipidaemia and obesity have been established. Abnormalities of fat metabolism, particularly triglyceride storage and lipolysis are an early manifestation of insulin resistance. Increased nonesterified fatty acid (NEFA) mobilization from adipose tissue leads to plasma NEFA increase and ectopic deposition of triglycerides in nonadipose tissues. Increased lipid deposition in muscle and liver contributes to the metabolic condition seen in type 2 diabetes, with increased lipid depositing in the pancreatic *β* cells contributing to *β*-cell dysfunction and accelerated apoptosis [35, 36]. In addition to its role as the principal storage depot of triglycerides, adipose tissue acts as an endocrine organ which contributes to whole-body energy homeostasis. The release of adiponectin, resistin and leptin during adipocyte differentiation regulates many aspects of lipid and glucose metabolism [37]. Adipocytes differentiate from preadipocytes (a progenitor population committed to an adipogenic lineage), in a process which requires multiple transcription factors, including master regulators PPAR*γ* (peroxisome proliferator-activated receptor gamma), C/EBPs (CAAT/enhancer-binding proteins) [38] and extracellular hormones including insulin [39, 40]. 

Esau et al. [41] were first to describe the role of miRNAs in human adipocyte biology. Using an adipocyte model system, the authors profiled the miRNA signatures of preadipocytes and differentiated adipocytes, which demonstrated that miR-143 was upregulated during differentiation. Inhibition of miR-143, with an antisense oligonucleotide inhibited differentiation, reduced the expression of adipocyte-specific genes (GLUT4, aP2, HSL, and PPAR-*γ*2) and decreased triglyceride accumulation (up to 75%) in treated cells in a dose-dependent manner. Efforts to further characterize the effects of miRNAs on mouse 3T3-L1 preadipocytes differentiation revealed that both miR-103 and miR-143 were unregulated during adipogenesis in vitro and in vivo [42]. Ectopic expression of miR-103, or miR-143 in preadipocytes, accelerated adipogenesis, increasing both the expression of adipogenesis markers and triglyceride accumulation. 

In 2006, Kajimoto et al. [43] studied the expression profile of miRNAs in 3T3-L1 preadipocytes during adipogenic differentiation. In this analysis, miR-10b, miR-15, miR-26a, miR-34c, miR-98, miR-99a, miR-101, miR-101b, miR-143, miR-152, miR-183, miR-185, miR-224, and let-7b were found to be upregulated, and miR-181a and miR-182 were downregulated during differentiation. These changes were not observed at early time points (days 1–5) but were observed at a relatively later stage of differentiation, during which time lipid droplets were visible (day 9). Use of antisense inhibition of differentially expressed miRNAs did not affect adipose differentiation; however, these miRNAs were thought to be involved in modulating adipocyte function.

Several miRNAs have been implicated in the regulation of adipogenesis through actions on key regulatory transcription factor PPAR*γ*. miR-27b is physiologically downregulated in human multipotent adipose-derived cells during adipogenesis. Overexpression of miR-27b was found to suppress differentiation by targeting a predicted miR-27b-binding site on PPAR*γ* mRNA, inhibiting expression of PPAR*γ* and downstream factor C/EBPa [44]. PPAR*γ* has also been found to be targeted and repressed by miR-130, which has a similar effect when overexpressed in human primary preadipocytes [45]. This demonstrates that miR-27b and miR-130 can regulate adipogenesis in these cells through direct activity on PPAR*γ* expression. Incidentally, thiazolidinediones, a class of insulin-sensitizing agents used in the treatment of insulin insensitivity, target PPAR*γ* to elicit their therapeutic effects [46]. PPAR*α* is another member of the PPAR family and important regulator of lipid homeostasis. PPAR*α* has been identified as a target of miR-519d and regulates the accumulation of lipid during adipogenic differentiation [47]. Manipulation of miR-519 expression in human visceral preadipocytes was found to modulate the differentiation of cells through changes in PPAR*α* expression. 

The miRNA paralogues miR-103 and miR-107 are thought to be involved in energy metabolism. These paralogues are highly conserved and located within the pantothenate kinase (PANK) gene. miR-103 genes generate two mature miRNAs, miR-103(1) and miR-103(2), while the miR-107 gene generates miR-107. PANK catalyses the rate limiting step of pantothenate phosphorylation during the generation of Coenzyme A (CoA), which is a critical cofactor of several enzymes involved in diverse metabolic pathways. Trajkovski et al. [48] investigated the connection between elevated expression of miR-103/107 and obesity in obese mice. In this study, they identified caveolin-1, a critical regulator of the insulin receptor, as a direct target gene of miR-103/107. Decreased levels of miR-103/107 in adipocytes increased caveolin-1 expression, which led to stabilization of the insulin receptor, enhanced insulin signalling, decreased adipocyte size, and enhanced insulin-stimulated glucose uptake. These findings demonstrate the central importance of miR-103/107 to insulin sensitivity.

The actions of miRNAs on insulin resistance in healthy and Goto-Kakizaki rats (a model of type 2 diabetes) were investigated by He et al. [49]. Insulin responsive tissues were characterized for their miRNA expression, which revealed highly elevated levels of miR-29 family in adipose, liver and skeletal muscle tissue isolated from each animal strain. The expression of miR-29 family genes was upregulated in diabetic tissues. This effect could be reproduced in vitro by incubating 3T3-L1 adipocytes in high glucose (hyperglycemia) and insulin (hyperinsulinemia), simulating a type 2 diabetic microenvironment. The action of these miRNAs on insulin signalling is not clearly understood. However, the authors identified that insulin-activated Akt activity was downregulated by miR-29. Insig1 (insulin-induced gene 1) and Cav2 (caveolin 2) were validated targets of miR-29; however, their role in insulin signalling was not understood. Ling et al. [50] furthered this work and identified 50 upregulated and 29 downregulated miRNAs in insulin-resistant adipocytes, including miR-320 which was expressed 50-fold higher in insulin-resistant cells. The authors found that miR-320 was able to regulate insulin resistance in resistant adipocytes through targeting of a kinase subunit, p85. This increased Akt phosphorylation and levels of Glut4, improving the insulin-PI3-K signalling pathways.

The majority of work characterizing miRNAs involved in insulin signalling and energy metabolism has involved single miRNAs (or paralogues) rather than complete clusters. Xu and Wong [51] completed a bioinformatic analysis screening for mouse signalling pathways targeted by miRNA clusters. The authors identified one miRNA cluster, mmu-mir-183-96-182 as a potential regulator of irs1, rasa1 and grb2, all of which are located in the insulin signalling pathway. It was theorized that a single miRNA cluster could act at multiple levels in a signalling cascade by targeting different components to control the signal transduction process.

## 4. miRNAs in Obesity

The medical profession and the wider community are very concerned by the increased health risks associated with the growing prevalence of overweight and obesity in developed cultures. Obesity is particularly significant for Australia, having recently overtaken the United States as the world's “fattest” nation. Many factors are contributing to the growing obesity around the world, but this can be primarily attributable to poor dietary habits and sedentary lifestyles conducive to developing the condition. 

Obesity, hyperlipidemia (elevated levels of blood lipids) and insulin resistance are strongly associated with the onset of type 2 diabetes. High Body Mass Index (BMI) has a strong correlation with increased (and excessive) adiposity which is principally responsible for a majority of the comorbidities associated with the condition. The activity of adipose tissue is adversely affected in obese patients, resulting in abnormal adipokine release and altered energy metabolism, which causes a state of chronic inflammation that contributes to insulin resistance and elevated free fatty acids in the bloodstream [52]. Obesity is most commonly associated with increased fat mass as a result of adipocyte hypertrophy or hyperplasia [37], which is closely linked to the chronic inflammation seen in patients. The elevated inflammatory cytokine levels typically observed in obese individuals, particularly TNF-*α* and IL-2 [53], secreted from adipocytes have been shown to impair adipogenesis, contributing to the detrimental deposition of lipids in other organs [54]. The relationship between obesity and diabetes is strongly related to glucose and lipid metabolism. miRNAs have been shown to regulate the activity of key cellular processes, including insulin release in pancreatic *β* cells and differentiation of adipocytes and are therefore thought to contribute to these pathologies. 

A consistent and counterintuitive observation in obese patients and experimental models of obesity is that the miRNAs normally induced during adipogenesis are downregulated in the obese subjects. Xie et al. [42] demonstrated this inversion in miRNA expression by profiling their expression in 3T3-L1 preadipocytes and adipocytes from leptin deficient ob/ob and diet-induced obese mice. Similar miRNAs were differentially regulated during in vitro and in vivo adipogenesis. miR-422b, miR-148a, miR-107, miR-103, miR-30c, miR-30a-5p, and miR-143 were induced during 3T3-L1 differentiation but downregulated in cells isolated from both models of obesity. This trend was also evident for miR-221 and miR-222, which were found to be downregulated during 3T3-L1 differentiation but upregulated in obese adipocytes. This inverse regulatory pattern has been observed during adipogenesis and obesity across different species, including mice and humans, and validated in different experimental models of obesity. 

As described previously, miR-143, miR-103 and miR-107 have been shown to regulate adipocyte differentiation. However, their expression has been found to be downregulated in the mouse model of genetic insulin resistance and obesity (ob/ob mice), possibly through an inflammatory pathway stimulated as part of the model pathology [42]. Visceral adipose tissue isolated from obese mice has been found to contain increased expression of miR-143, which is associated with alterations to PPAR*γ* and aP24 expression [55]. Chronic inflammation is typically seen in obesity and is associated with changes to the miRNA profile of affected tissues. In three murine models of obesity including leptin-deficient ob/ob mice, leptin-receptor-deficient db/db mice, and KKAy44 mice, miR-335 was found to be upregulated in adipose tissue [56], suggesting a role in adipose hyperplasia, despite the lack of experimentally validated targets of miR-335 in adipose tissue. 

In a recent study, Zhao et al. [57] profiled 220 miRNAs in pancreatic islets, adipose tissue and liver isolated from diabetes-resistant (B6) and diabetes-susceptible (BTBR) mice. Many miRNAs in these tissues were altered following induction of obesity, with several miRNAs in each tissue responding differently, depending on the animal strain. Approximately 40 miRNAs in liver were downregulated in response to obesity in diabetes-resistant mice but not in diabetes-susceptible animals, indicating that genetic differences between the mouse strains play a critical role in miRNA regulation and illustrating the possible contribution of genetic background to miRNA expression in disease progression. 

In 2009, Klöting et al. [58] isolated miRNA from different fat depots of overweight and obese individuals. Paired samples of abdominal subcutaneous and intra-abdominal omental adipose tissue that were obtained from fifteen individuals with either normal glucose tolerance or recently diagnosed type 2 diabetes were analysed. Analysis revealed that of the 106 miRNAs expressed in these tissues, there was no miRNA exclusively expressed in either fat depot. However, 16 miRNAs had an expression pattern dependent on the fat depot. The expression of miRNA-17-5p, miRNA-132, miRNA-99a, miRNA-134, miRNA-181a, miRNA-145 and miRNA-197 were correlated with key metabolic parameters, including, fasting plasma glucose, and circulating leptin, adiponectin and interleukin-6 levels. A negative correlation between miR-99a, miR-325 and IL-6 concentration was determined, as was an inverse correlation between miR-181a expression and adiponectin concentration.

## 5. miRNAs in Clinical Applications

miRNAs represent a possible diagnostic tool for the early detection of type 1 diabetes. Patients suffering from the disease will have already lost more than 50% of their *β*-cell mass before presenting with symptoms. There is therefore a need to develop a system for early detection, to allow intervention strategies to be significantly more effective. miRNAs have been detected in blood and other body fluids [59] and miRNAs isolated from serum samples have proven to be stable, reproducible and consistent among individuals [60, 61]. In a recent study, Zampetaki et al. [62] extracted miRNAs from plasma samples of age- and sex-matched type 2 diabetic patients and controls. They determined 13 candidate miRNAs with microarray screening that were differentially expressed in these samples. Quantitative PCR assessment revealed lower plasma levels of miR-20b, miR-21, miR-24, miR-15a, miR-126, miR-191, miR-197, miR-223, miR-320, and miR-486 and an increase in miR-28-3p in diabetic serum. Interestingly, the expression of miR-15a, miR-28-3p, miR-126, miR-223 and miR-320 was altered before the disease was manifested clinically. Analysis of these miRNAs was sufficient to identify 70% of the type 2 diabetic patients and predict the development of type 2 diabetes in 52% of normoglycemic patients that developed diabetes over the course of the study. This was the first demonstration that a unique miRNA blood signature could exist for diabetes and when combined with other clinical information could distinguish between patients with prevalent or incident diabetes from healthy controls.

The nature of the interactions between miRNAs and the 3′-UTRs of mRNA affords a single miRNA with the potential to post-transcriptionally regulate the expression of a wide pool of target genes. This makes miRNAs a prime target for direct therapeutic intervention. Altering the function of a single miRNA could significantly alter the behaviour of treated cells. In many disease conditions, the expression of miRNAs can be significantly altered. Therefore, pharmacologically returning miRNA expression to normal levels could improve clinical outcomes and improve current therapies. miRNA levels can be experimentally reduced in vivo using antisense oligonucleotide miRNAs (antimiRs). Esau et al. [63] demonstrated the potential therapeutic effects of inhibition of liver miR-122 in mice by using 2′-*O*methoxyethyl phosphorothioate-modified oligodeoxy-nucleotides. miR-122 inhibition in a diet-induced obesity mouse model resulted in decreased plasma cholesterol levels, a significant improvement in liver steatosis, and suppression of several lipogenic genes. This study validated this strategy as a novel means to regulate energy metabolism, such as, cholesterol and fatty-acid metabolism in the adult liver. In their study of glucose metabolism in multiple organs, Frost and Olson [64] demonstrated that global knockdown of the Let-7 family with an antimiR was sufficient to prevent and treat impaired glucose tolerance in mice with diet-induced obesity. This effect was thought to be attributable to increased insulin sensitivity in liver and muscle tissue. The antimiR treatment on these mice resulted in increased lean and muscle mass with stable fat mass and prevented ectopic fat deposition in the liver. Their findings demonstrate the potential benefits of miRNA manipulation in the context of insulin resistance, with the Let-7 family representing a potential therapeutic target for the treatment of type 2 diabetes. 

The alternative to this technology involves the use of miRNA mimics or mimetics, which can be used to pharmacologically increase the levels of a particular miRNA [65]. As described previously, a consistent and counterintuitive observation in obese patients and experimental models of obesity is that the miRNAs normally induced during adipogenesis are downregulated in the obese subjects [42]. It is therefore unlikely that this technology will be applicable in the context of insulin resistance, as increased expression of disregulated miRNAs would most likely exacerbate, rather than treat the condition. However, this does not mean that this technology is not applicable in other experimental or therapeutic applications. The use of miRNA mimics in a therapeutic context has been demonstrated in a mouse model of nonsmall cell lung cancer [66]. A miR-34a mimic, in a lipid-based vehicle, was able to induce apoptosis and significantly inhibit tumour growth. 

Current studies that experimentally alter the activity of miRNAs to elicit a particular biological effect are very limited, with researchers typically confined to altering the expression of a particular family (such as the Let-7 family) or a specific miRNA to monitor its effects. These studies represent fundamental experiments that will determine the clinical potential of therapies utilizing miRNA manipulations. The technology necessary to manipulate multiple miRNAs in a clinical context does not presently exist. However, it is possible that current techniques used to manipulate limited miRNA expression could be adapted for use with conventional treatments. This would represent a class of novel therapies, combining traditional therapeutics with miRNA manipulation technologies to improve clinical outcomes. In the context of insulin resistance, manipulation of only a few specific miRNAs could potentially improve the efficacy of or suppress development of tolerance to pharmaceuticals traditionally used to improve glucose tolerance and insulin sensitivity. In the future, characterization of the miRNA signature in healthy and diseased individuals would facilitate the design of a therapy to restore the healthy expression of an entire repertoire of disregulated miRNAs in the disease state. With the introduction of Personalized Medicine [67], this therapy would also become highly specific, tailored to the miRNA signature of an individual, allowing for very precise and effective treatment.

## 6. Conclusions

Diabetes and obesity have emerged as one of the greatest challenges faced by contemporary medicine. While motivated patients may overcome type 2 diabetes and obesity through managed life-style changes, current therapies are otherwise limited to restricting disease progression. miRNAs have the potential to regulate the expression of a wide spectrum of target genes, making these small molecules very valuable therapeutic targets (see Table 1). However, it is only through continued investment in the study of miRNAs in metabolic health and disease that their therapeutic potential can be fully realized. 

## Figures and Tables

**Table 1:** Summary of key biological processes and miRNAs discussed in this paper.

Biological process	Specific miRNAs	References
ngn3-independent endocrine pancreas regeneration	miR-15a, miR-15b, miR-16 and miR-195	[10]

Regulates insulin expression	miR-30d, miR-375, miR-124a2	[17, 22, 30]

Regulates insulin secretion	miR-375, miR-9, miR-124a2	[8, 25, 31]

Regulates glucose-stimulated insulin secretion (GSIS)	miR-369-5p, miR-130a, miR-27a, miR-410, miR-200a, miR-337, miR-532, miR-320, miR-192 and miR-379, miR-375, miR-124a2	[18, 20, 21, 32]

Regulates adipocyte differentiation	miR-143, miR-27b, miR-130, miR-519d	[41, 42, 44, 45, 47]

Regulates insulin sensitivity	miR-103, miR-107, miR-29, miR-320, mmu-mir-183-96-182 (cluster)	[48–51]
